# Supplementary material for: Triglyceride and Glucose Index as an Optimal Predictor of Metabolic Syndrome in Lebanese Adults
Source: Nutrients. 2024 Oct 30;16(21):3718. doi: 10.3390/nu16213718 (PMC11547298; doi:10.3390/nu16213718)
Supplement: Supplementary file 1 [file nutrients-16-03718-s001.zip › nutrients-3266308-supplementary.pdf]

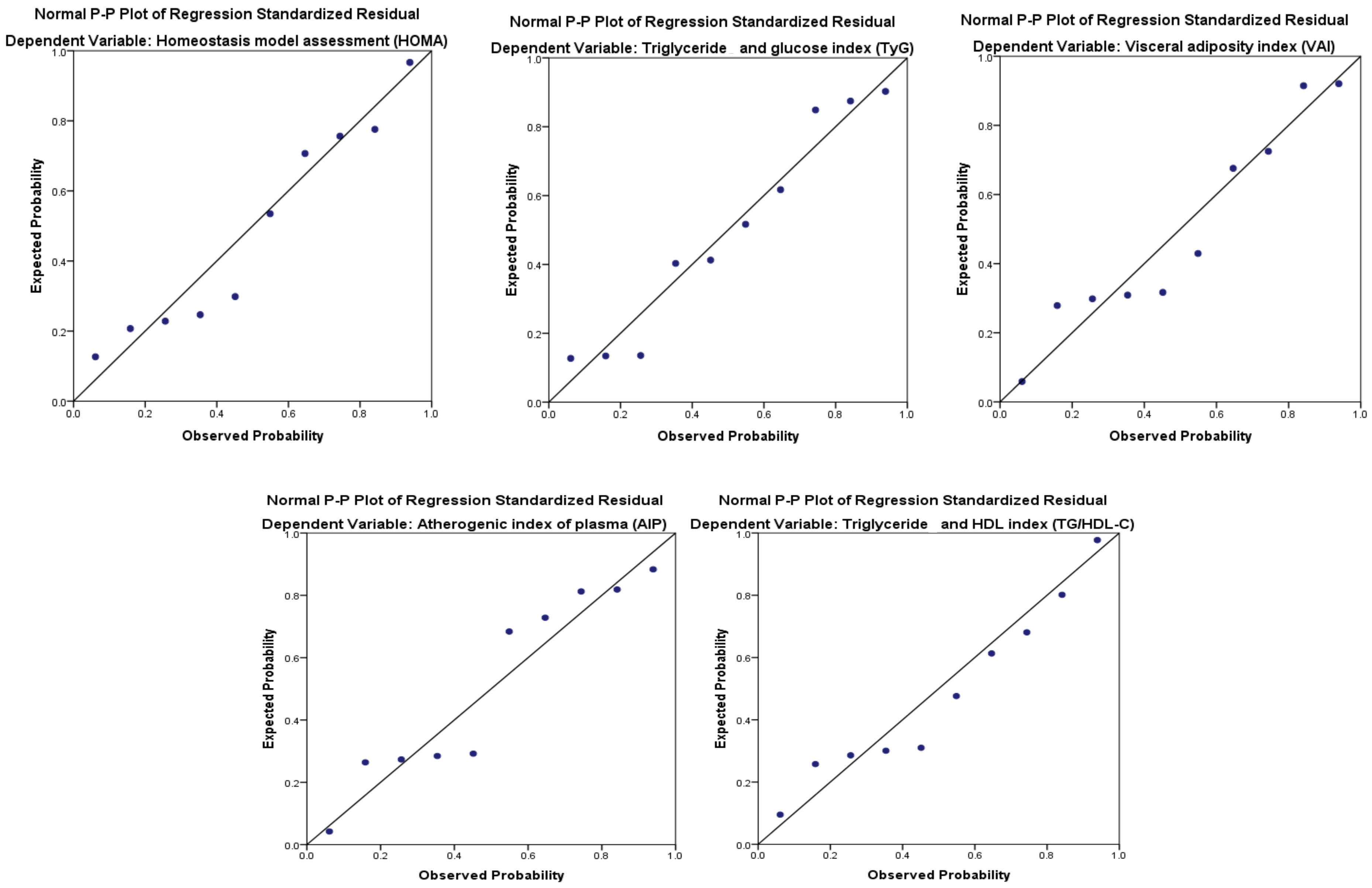

Figure S1: Calibration plots of the biochemical parameters to predict the metabolic syndrome based on the contingency table for Hosmer-Lemeshow statistic in females.

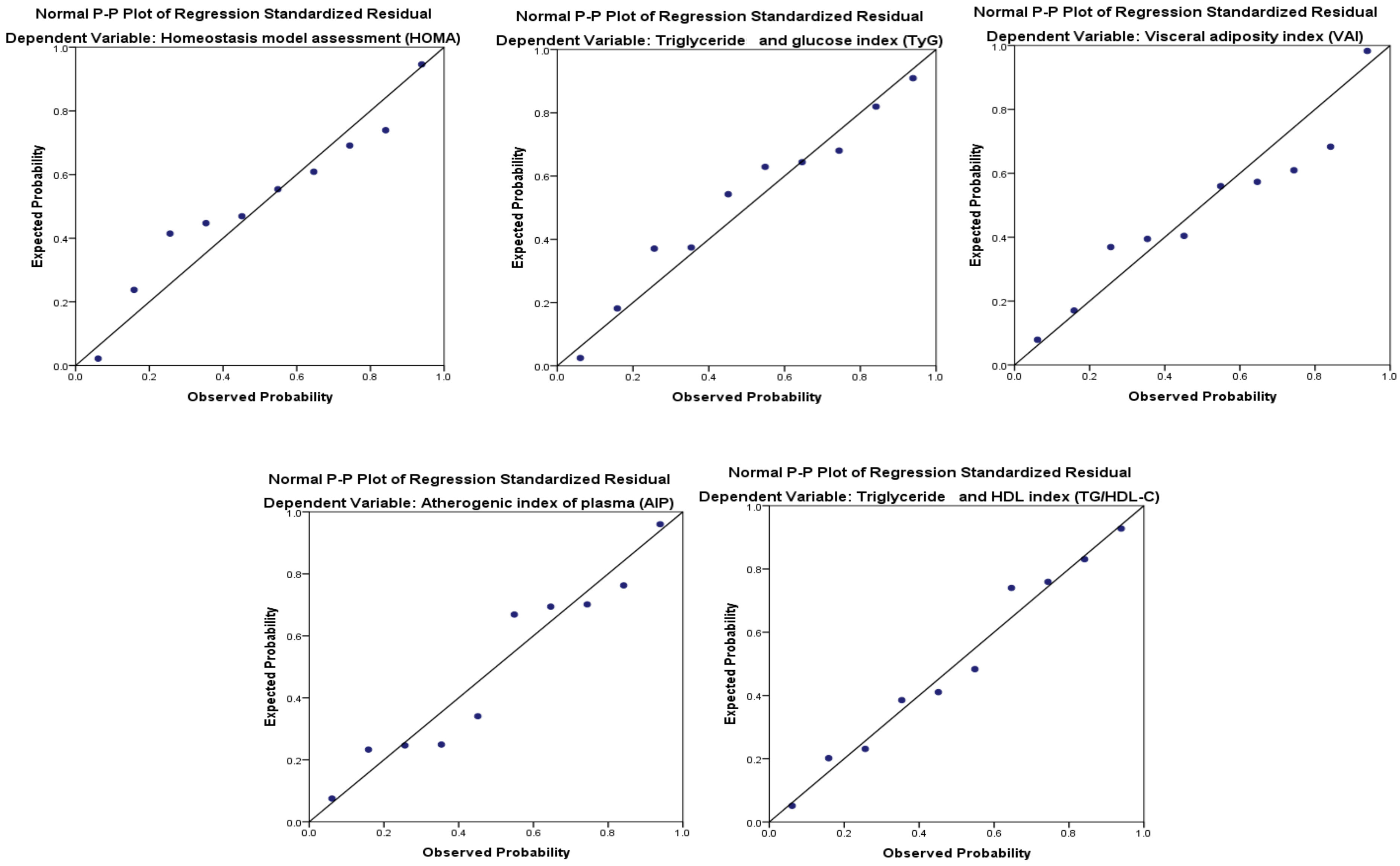

Figure S2: Calibration plots of the biochemical parameters to predict the metabolic syndrome based on the contingency table for Hosmer-Lemeshow statistic in males.

Table S1: The AUC of biochemical parameters for the presence of the Metabolic Syndrome

	Total sample AUC (95% CI)	Females AUC (95% CI)	Males AUC (95% CI)
Homeostasis model assessment (HOMA)	0.71 (0.64; 0.77)	0.71 (0.62; 0.80)	0.75 (0.64; 0.85)
Triglyceride and glucose index (TyG)	0.87 (0.82; 0.91)	0.84 (0.77; 0.90)	0.91 (0.84; 0.97)
Visceral adiposity index (VAI)	0.83 (0.78; 0.88)	0.82 (0.75; 0.89)	0.89 (0.83; 0.96)
Atherogenic index of plasma (AIP)	0.84 (0.78; 0.89)	0.80 (0.72; 0.87)	0.88 (0.80; 0.95)
Triglyceride and HDL index (TG/HDL-C)	0.84 (0.78; 0.89)	0.80 (0.72; 0.87)	0.88 (0.80; 0.95)
